# Supplementary material for: Temporal Dynamics of Cytokine, Leukocyte, and Whole Blood Transcriptome Profiles of Pigs Infected with African Swine Fever Virus
Source: Pathogens. 2025 Oct 1;14(10):992. doi: 10.3390/pathogens14100992 (PMC12567412; doi:10.3390/pathogens14100992)
Supplement: Supplementary file 1 [file pathogens-14-00992-s001.zip › Figure S1 Gating strategies for flow cytometry.pdf]

Figure S1: Gating strategies for flow cytometry.

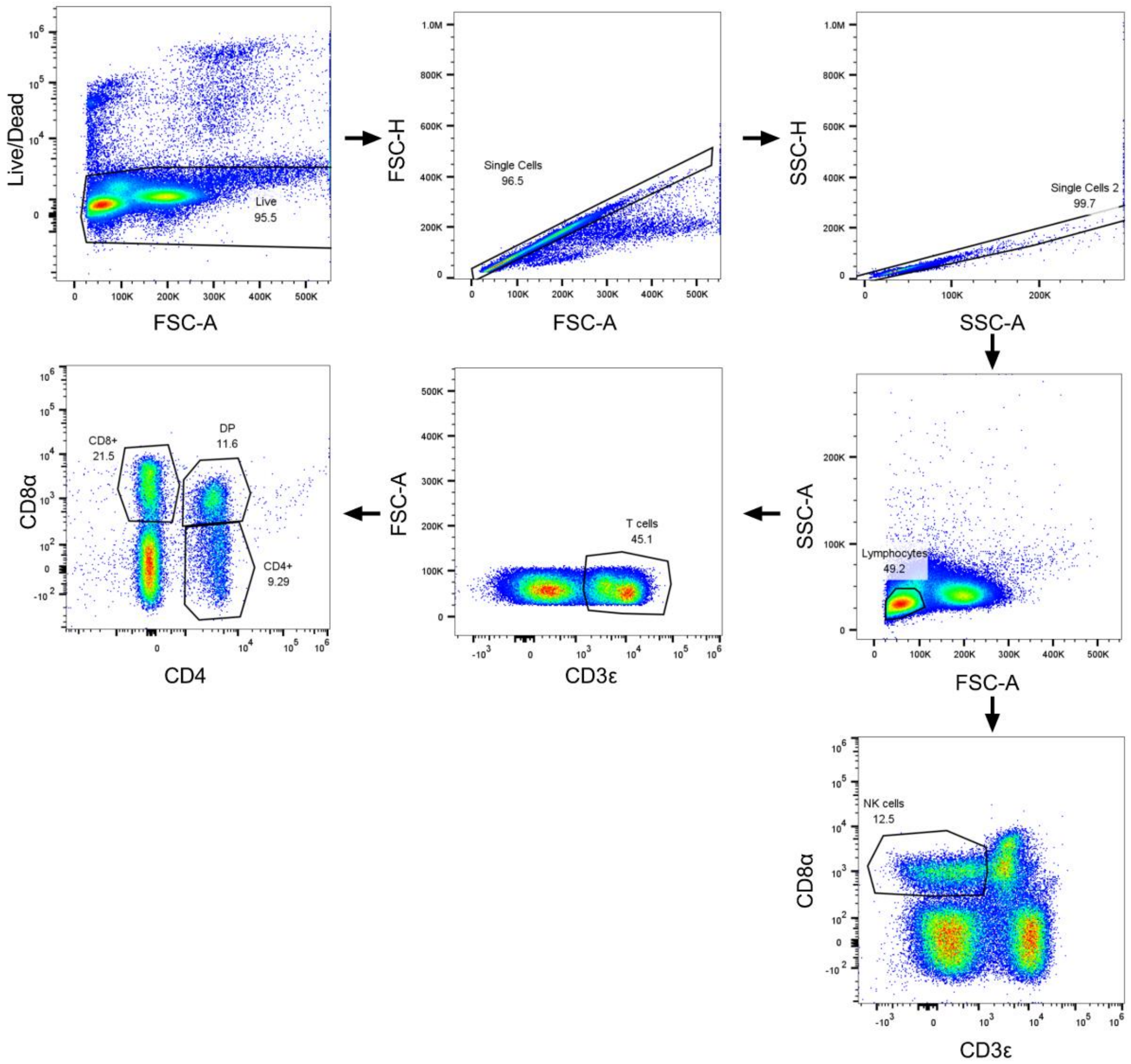

T and NK Cell Panel – Gating

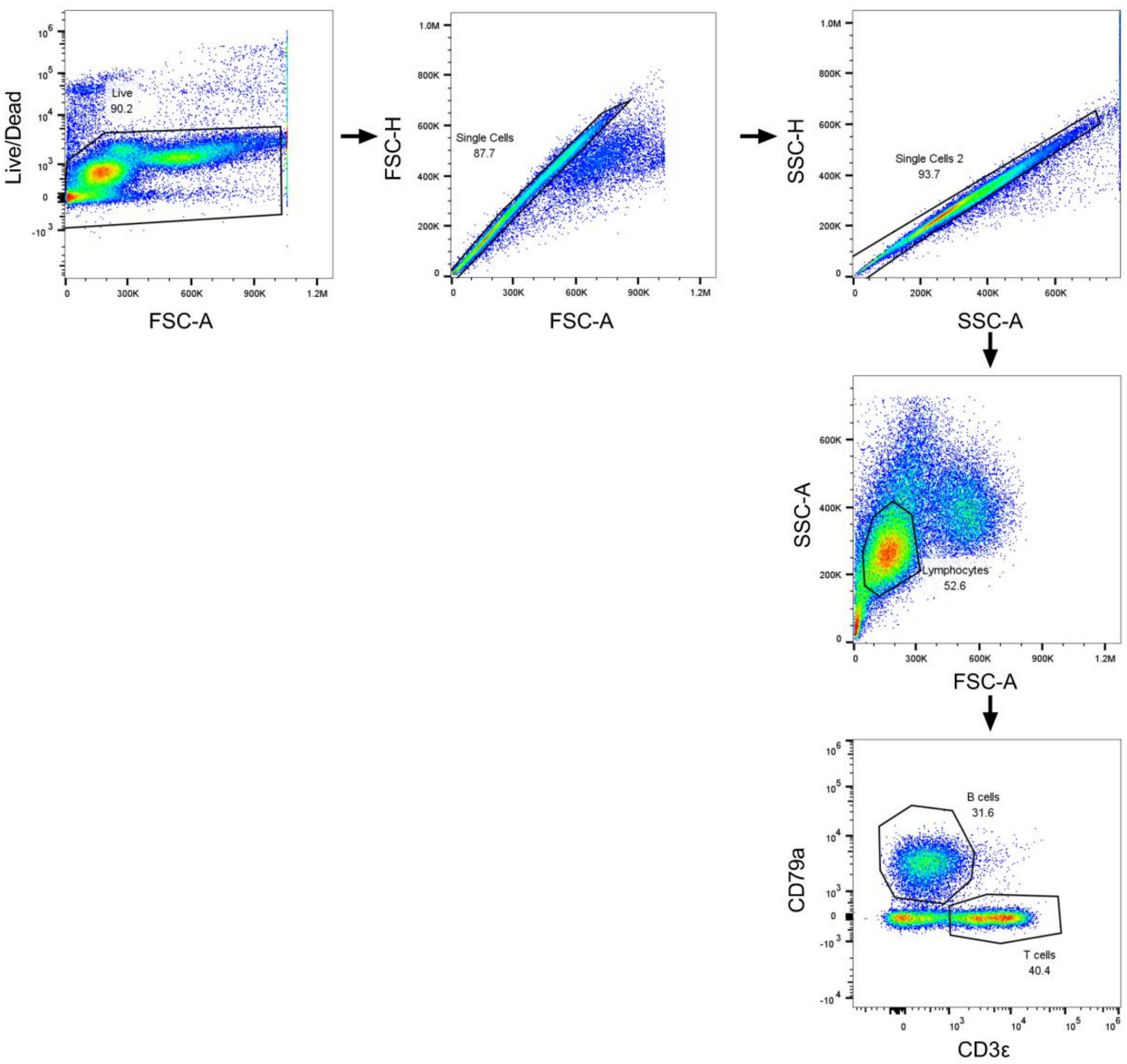

B and T Cell Panel – Gating

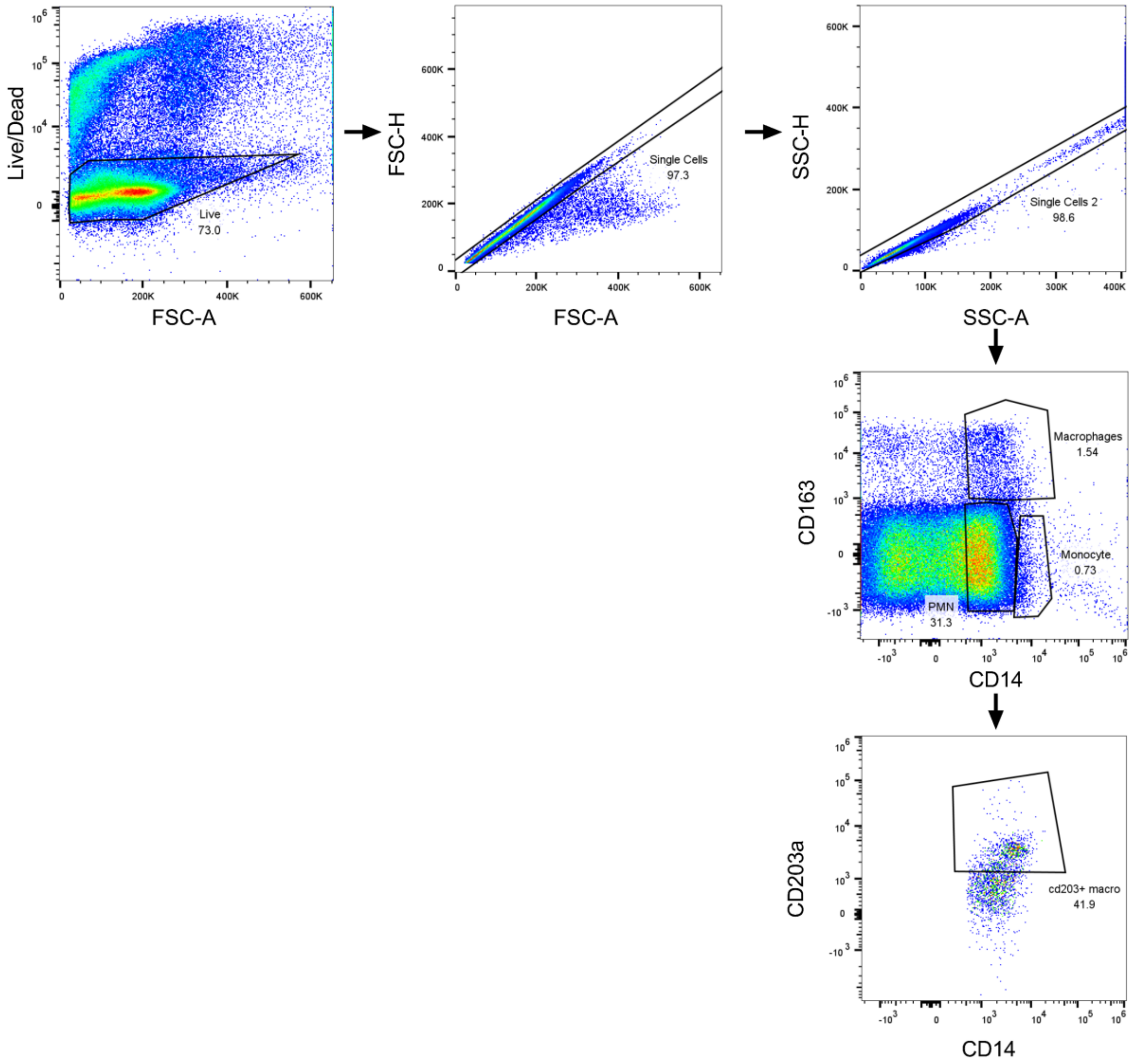

Monocyte Macrophage Panel Gating
